# Strain-modulated ferromagnetism and band gap of Mn doped Bi_2_Se_3_

**DOI:** 10.1038/srep29161

**Published:** 2016-07-04

**Authors:** Shifei Qi, Hualing Yang, Juan Chen, Xiaoyang Zhang, Yingping Yang, Xiaohong Xu

**Affiliations:** 1 School of Chemistry and Materials Science, Shanxi Normal University, Linfen, Shanxi 041004, China

## Abstract

The quantized anomalous Hall effect (QAHE) have been theoretically predicted and experimentally confirmed in magnetic topological insulators (TI), but dissipative channels resulted by small-size band gap and weak ferromagnetism make QAHE be measured only at extremely low temperature (<0.1 K). Through density functional theory calculations, we systemically study of the magnetic properties and electronic structures of Mn doped Bi_2_Se_3_ with in-plane and out-of-plane strains. It is found that out-of-plane tensile strain not only improve ferromagnetism, but also enlarge Dirac-mass gap (up to 65.6 meV under 6% strain, which is higher than the thermal motion energy at room temperature ~26 meV) in the Mn doped Bi_2_Se_3_. Furthermore, the underlying mechanisms of these tunable properties are also discussed. This work provides a new route to realize high-temperature QAHE and paves the way towards novel quantum electronic device applications.

Topological insulators (TIs) have attracted tremendous attention because of its robust surface states, which are protected by time-reversal symmetry (TRS) and induced by strong spin-orbit coupling (SOC)^1^,^2^,^3^,^4^,^5^,^6^,^7^,^8^. In particular, there is great interest in the effect of magnetic doping, which is considered to be an efficient way to open an energy gap in the Dirac cone by breaking TRS^9^,^10^,^11^, resulting in the emergence of massive Dirac fermions. This gap is expected to give rise to many interesting phenomena, such as Majorana fermion^12^, topological magnetoelectric effect^13^. Especially in a TI thin film, ferromagnetic order can also be established, which potentially could realize the quantum anomalous Hall state (QAHE)^14^,^15^.

Breaking the TRS of a suitable TI film by introducing ferromagnetism can naturally lead to the QAHE^15^. But, a crucial issue in the realizing of QAHE is how to obtain magnetic and insulated TIs. A conventional strategy to produce magnetism in TIs is by magnetic doping, similar to diluted magnetic semiconductors (DMS)^16^. Ferromagnetic TIs via magnetic doping have been reported in the past few years, including Fe doped Bi_2_Te_3_^9^,^17^, V, Cr and Mn doped Sb_2_Te_3_^18^, and Mn and Cr doped Bi_2_Se_3_^19^,^20^. But, QAHE has been challenging to realize in transition metal doped TIs. Recently, the first experimental observation^21^ and further approve of QAHE in Cr or V doped (Bi, Sb)_2_Te_3_ have been reported^22^,^23^,^24^. A zero field Hall resistance exhibits a distinct plateau with the quantized value h/e^2^ in these experiments, which confirms the existence of the QAHE. However, as shown in their experiments, above 1 K, dissipative channels from surface states and bulk bands are thermally activated, which can smear out the transport properties of the QAHE edge states. In addition, low Curie temperature also influences the observation of QAHE. Hence, strong ferromagnetic TIs with large-size band gap need to be developed in order to realize high temperature QAHE, which will be the prerequisite to the development of low-power-consumption electronics based on QAHE in TIs.

It is well known that strain is a very important factor from the film growth and application aspect of nanodevices. This is because that strain is not avoidable during epitaxial growth if there is a lattice mismatch between grown nanostructure and substrate. Additionally, strain has also become a routine factor to engineer band gaps and influence some important properties such as magnetism and so on^25^,^26^. Recently, Strain modulated topological insulator properties of graphene/TI hybrid systems are also presented in the some works^27^,^28^. Here, we make a systematically study of the strain effects on magnetic property and electronic structures of Mn doped Bi_2_Se_3_ using first-principles calculations. We first show that the energetic stability and ferromagnetism of Mn doped Bi_2_Se_3_ can be tuned by out-of-plane tensile strain. Furthermore, out-of-plane tensile strain can enlarge the Dirac-mass gap of Mn doped Bi_2_Se_3_. The mechanisms of these tunable properties are also analyzed.

## Results and Discussion

**Figure 1:**
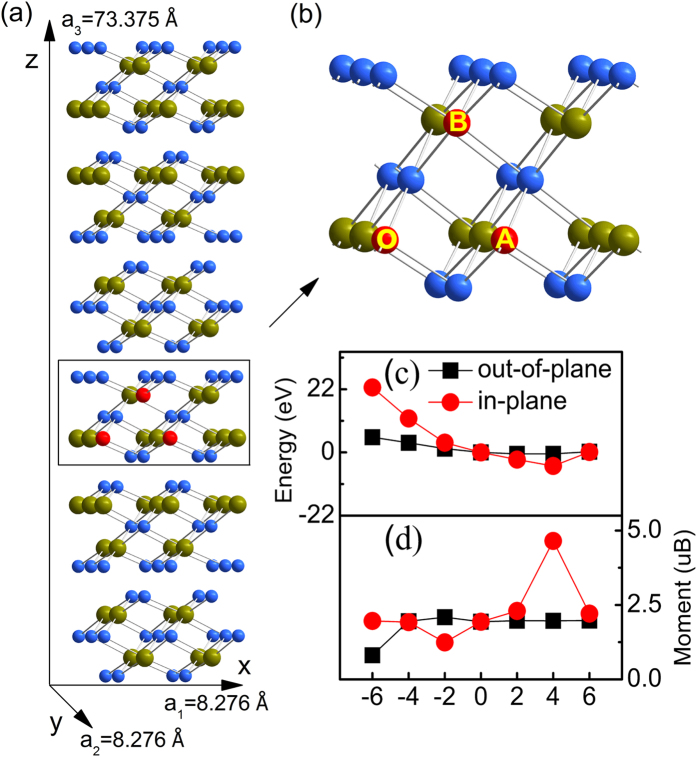
Energetics and magnetic behavior of Mn doped Bi_2_Se_3_ under strain. (**a)** A 2 × 2 × 1 supercell of 6QLs Bi_2_Se_3_ used in the calculation. **(b)** Only 1 of 6QLs is used to clarity the doped configurations. One Mn is fixed at O and the others move from A to B. **(c)** Total energies relative to the case without strain versus in-plane and out-of-plane strains. **(d)** Moments versus in-plane and out-of-plane strains.

We start from studying the effect of strain on stability of Mn doped Bi_2_Se_3_. Experiments^18^,^20^ and theoretical calculations^29^,^30^ have suggested that the magnetic dopants, such as V, Cr, Mn and Fe, will mostly substitute the Bi or Sb ions in 3D TIs; we therefore concentrate on this situation. As shown in Fig. 1c, the total energies of Mn doped Bi_2_Se_3_ with different strains are presented. The total energy of Mn doped Bi_2_Se_3_ without strain is set as zero, namely the energies of Mn doped Bi_2_Se_3_ with strains are given relative to the case without strain. The positive and negative strains respectively indicate the tensile and compressive lattice constants. When compressive strains are imposed, whether in-plane or out-of-plane strains, it usually decreases the stability of Mn doped Bi_2_Se_3_, however, the opposite situation happens for the tensile strain. From Fig. 1c, we can find that the Mn doped Bi_2_Se_3_ with in-plane and out-of-plane tensile strains become more stable than that without strain. And the stability of studied system increases with strength of tensile strains (2% to 4%). With the tensile strain up to 6%, the stability of Mn doped Bi_2_Se_3_ is the same as that of the case without strain. In the following discussion, we will find that this change in stability is related to the difference of electronic transport in Mn doped Bi_2_Se_3_ with different tensile strains. Hence, our results show that modest in-plane or out-of-plane tensile strains will make Mn doped Bi_2_Se_3_ becomes more stable. In the following discussion, we will find that this is because that strain has effect on the magnetic property of the Mn doped Bi_2_Se_3_, which furthermore change the relative stability of the Mn doped Bi_2_Se_3_ with and without strain.

**Figure 2:**
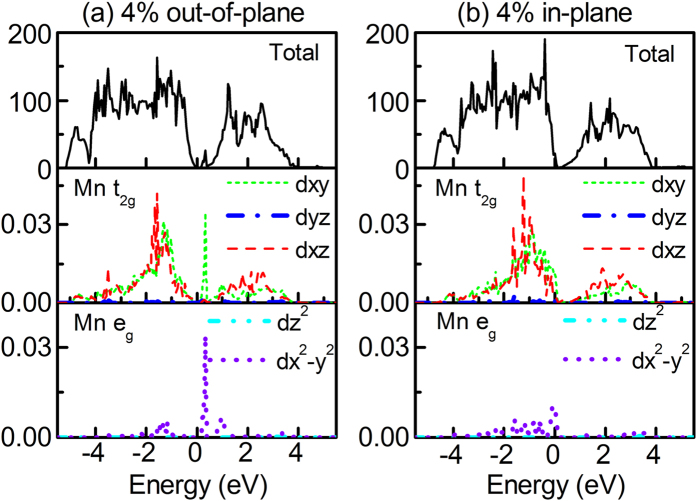
Total and partial densities of states of Mn doped Bi_2_Se_3_. (**a**) with 4% out-of-plane. (**b**) 4% in-plane strains.

It is more interesting to observe that the magnetic property is modulated with strain, especially for tensile strain. In this study, we focus on the influence of strain on magnetic moment and ferromagnetic stability of Mn doped Bi_2_Se_3_. Firstly, in Fig. 1d, we provide the change in magnetic moment of Mn doped Bi_2_Se_3_ with strain. For the most of Mn doped Bi_2_Se_3_ with strain, the moment nearly does not alter except those with −6% out-of-plane, −2% in-plane, and 4% in-plane strains. For example, the moment of the case with 4% in-plane strain is about 4.8 μB, which is almost twice larger than the case with 4% out-of-plane strain. The change in magnetic moment is usually related to the different *d* electrons occupation, which have been found that strains can change the *d* electrons occupation^31^. To show this is indeed the case. In Fig. 2, we compare the densities of states (DOSs) of Mn doped Bi_2_Se_3_ under 4% out-of-plane strain with the case under 4% in-plane strain. The most obvious difference in Fig. 2a,b is the appearance of various degeneracy of Mn 3*d* orbitals under 4% out-of-plane and in-plane strains. For the case with 4% out-of-plane strain, the 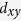, 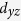 and 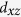 orbitals is degenerated and form three fold-degeneracy 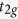 state, while 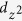 and 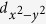 orbitals form twofold-degeneracy 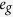 state. When 4% in-plane strain is applied, degeneracy of Mn 3*d* orbitals is fivefold, which results in larger moment.

**Figure 3:**
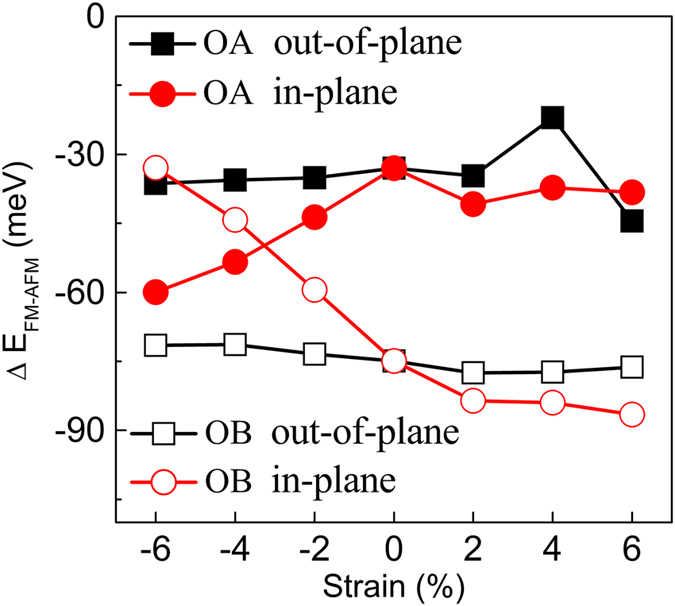
Magnetic coupling between two Mn atoms versus in-plane and out-of-plane strains in the OA and OB configurations. Configurations are shown in the Fig. 1b.

To see whether ferromagnetism can also be tuned by strain in the Mn doped Bi_2_Se_3_, we next study the magnetic interaction between two Mn ions. As indicated in Fig. 1b, we have considered two kind of different Mn substitution sites (One Mn atom substituting a Bi at O is fixed and the other moves from A to B): in-plane (OA) and out-of-plane (OB) configurations. The magnetic interaction between the two Mn dopants at a given separation is evaluated by calculating the total energy difference between the ferromagnetic (FM) and the antiferromagnetic (AFM) configurations (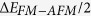) of the two Mn moments. The results are summarized in Fig. 3. Our results reveal that the magnetic coupling of OA and OB configurations always favors FM state under out-of-plane and in-plane strains. The FM stability of OA configuration is in the range of −60 to −20 meV under different strains. However, for the OB configuration with different strain, the FM stability (except −4% and −6% in-plane strains) is generally stronger (about −60 to −85 meV) than that in OA configuration. We find that the distance of two doped Mn atoms are respectively 3.97 and 3.89 Å in the OA configurations with −4% and −6% in-plane strains. While they are 4.40 and 4.37 Å in the OB configurations with −4% and −6% in-plane strains. Therefore, smaller Mn-Mn distance leads to stronger FM coupling in the OA configuration than that in the OB configuration. For the other cases with different in-plane strains from −2% to 6%, the magnetic moments are larger than those in the OA configuration. Thus, FM stability in OB configuration is stronger than that in the OA configuration. We have also calculated the Curie temperature under different strains within the mean-field theory (see Supplementary Fig. S1 and corresponding discussion). Usually, the *T*_c_ from the mean-field theory is larger about 50% than that from Monte Carlo simulation^32^. Hence the *T*_c_ of Mn doped Bi_2_Se_3_ with different strains should be in the range of 30–150 K at 4.16% Mn doping. The magnetic anisotropy energy is also important to the QAHE. We have obtained the magnetic anisotropy energy (MAE) of A and B configurations. As listed in the Supplementary Table S1, the results show that out-of-plane ferromagnetism is more stable than in-plane one. This result agrees with experimental finding from Xu *et al*.^20^. Additionally, the FM strength can be strengthened with the change from compressive strain to tensile strain for this OB configuration. Hence, the ferromagnetic stability and tunable regularity by strain in OB configuration is better than that in OA configuration. Furthermore, we can see that tensile strains will make FM stronger than that without strain in OB configuration. Thus, we can say that modest in-plane or out-of-plane tensile strains will make the ferromagnetism of Mn doped Bi_2_Se_3_ become stronger.

**Figure 4:**
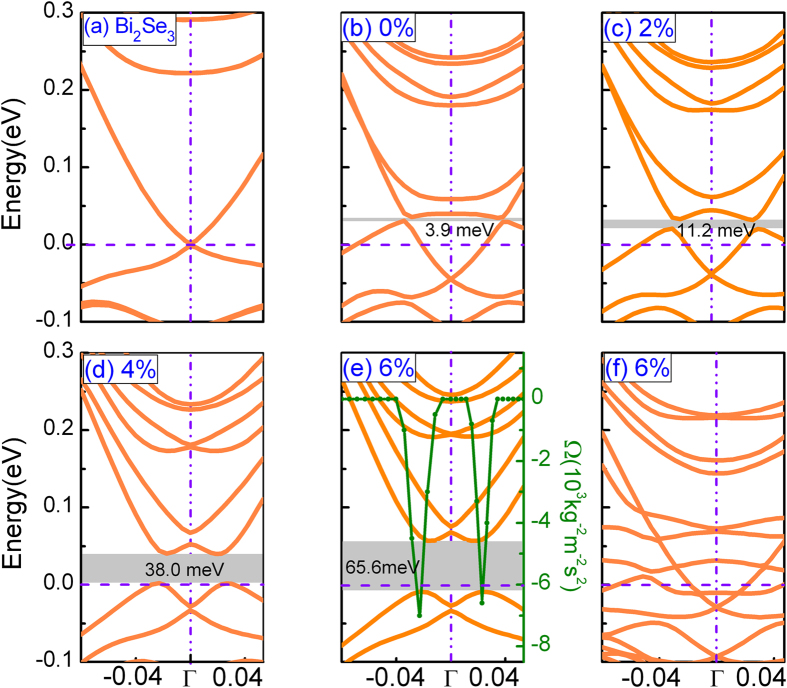
Band structures of Mn doped Bi_2_Se_3_ under strain. **(a)** pure Bi_2_Se_3_, **(b)** Mn doped Bi_2_Se_3_ without strain, **(c–e)** Mn doped Bi_2_Se_3_ with different out-of-plane tensile strains, and **(f)** Mn doped Bi_2_Se_3_ with 6% in-plane tensile strain. Circle symbols: corresponding Berry curvatures along the high-symmetry directions.

Above results have confirm that modest in-plane or out-of-plane tensile strains are propitious to the stability and FM strength of Mn doped Bi_2_Se_3_. We then turn to see the influence of in-plane or out-of-plane tensile strains on the band structures of the Mn doped Bi_2_Se_3_. Figure 4a shows the band structure calculated by using a 2 × 2 slab supercell of the ideal Bi_2_Se_3_ surface with 6 QLs thick. As can be seen, the Fermi level (E_f_) is just at the Dirac point, which is in agreement with previous results^33^,^34^ and indicates that computational method used in this study is reasonable. After Mn is doped into Bi_2_Se_3_ (Fig. 4b), a tiny Dirac gap about 3.9 meV is opened. If out-of-plane tensile strain is applied in Mn doped Bi_2_Se_3_, the Dirac-mass gap increases from 11.2 to 65.6 meV with strains as displayed in Fig. 4c-4e. Hence, the Dirac-mass gap 65.6 meV at 6% out-of-plane tensile strain is larger than that 27 meV i.e. band gap of room-temperature thermal exicition. But, in the case of in-plane tensile strain, for example 6%, our results show that the Dirac gap disappears, as shown in Fig. 4f. In fact, we calculate the band structures of Mn doped Bi_2_Se_3_ with different (from 2% to 6%) in-plane tensile strains, no Dirac gap is opened. In addition, we find that E_f_ is also shifted into the Dirac gap under 6% out-of-plane strain. This indicates that we can modulate the Dirac gap and E_f_ by out-of-plane tensile strain.

We have calculated the Berry curvature of the OB configuration with 6% out-of-plane tensile strain. The corresponding results are shown in the Fig. 4e. The Berry curvature is obtained by integrating the occupied valence bands using the expression^35^,^36^


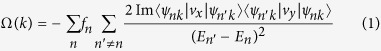


where *n* is the band index, *E*_n_ and 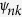 are the eigenvalue and eigenstate of the band *n*, 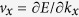 and 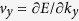 are the velocity operators along the *x* and *y* directions within the film plane, and *f*_*n*_ = 1 for all *n* occupied bands. This integration gives the corresponding Hall conductance^36^. The Berry curvature distribution along the high symmetry directions shows a large negative peak near the Γ point and zero elsewhere else (See Fig. 4e). As a consequence, the total integration or the Hall conductance with the Fermi level lying inside the band gap must be nonzero. The system should be in a QAH state. In addition, in order to confirm the stability of Mn doped Bi_2_Se_3_ under 6% out-of-plane tensile strain, we also perform the calculation of phonon dispersion curves Mn doped Bi_2_Se_3_ under 6% out-of-plane tensile strain in a 6QLs 1 × 1 supercell(see Supplementary Fig. S3). It can be found that 6% out-of-plane tensile strain will not lead to any imaginary frequency dispersion for the Mn doped Bi_2_Se_3._ Thus, this system should be stable in the 6% out-of-plane tensile strain.

**Figure 5:**
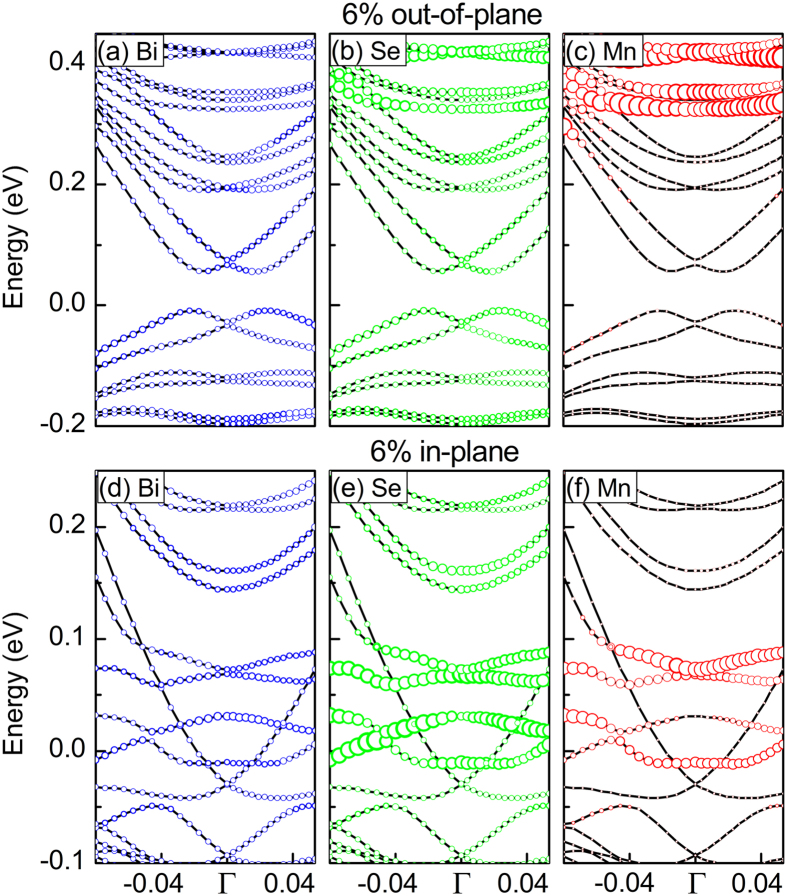
The character of band structures of Mn doped Bi_2_Se_3._ **(a)**with 6% out-of-plane strains. **(b)** Mn doped Bi_2_Se_3_ with 6% in-plane strain, obtained by projecting Kohn-Sham states to the local orbitals on a single atom of each type.

Why can the out-of-plane tensile strain tune the Dirac gap, and why can not in-plane tensile strain Dirac gap in Mn doped Bi_2_Se_3_? From previous study, FM exchange field can open surface gap in TIs^14^. In detail, lots of studies have proved that Bi_2_Se_3_ is a TI system. Thus, Bi_2_Se_3_ must be topologically nontrivial one. In such system, band structures are inverted and cross at the Dirac point (no gap is opened). And then if a sufficiently large exchange field is involved, the band inversion will be increased and leads to a topologically nontrivial band gap opened. Thus, for the Mn doped Bi_2_Se_3_ without any strain, a small Dirac gap is opened due to ferromagnetic exchange field. Our above results have shown that in-plane and out-of-plane tensile strains can strengthen ferromagnetism of Mn doped Bi_2_Se_3_. It is reasonable that Dirac gap increases with out-of-plane tensile strains in Mn doped Bi_2_Se_3_. But, from Fig. 3, we can find that the ferromagnetism of OB configuration with in-plane tensile strain is stronger than that with out-of-plane tensile strain. The Dirac gap should become larger in the case of in-plane tensile strain. Then, why is Dirac gap in Mn doped Bi_2_Se_3_ closed under in-plane tensile strain? In order to understand this difference, we provide band contribution analysis from different elements for the Mn doped Bi_2_Se_3_ with 6% out-of-plane and in-plane tensile strains in Fig. 5. For the case with out-of-plane tensile strain, in Fig. 5a, it can be found that valence bands close to the Fermi level are mainly dominated by Bi and Se atoms, while Mn contributes mainly to the lower valence and higher conduction bands farther away from the Dirac gap. While the in-plane tensile strain is applied, from Fig. 5b, we can see that lots of hybrid states leaded by Mn, Se, and Bi will occupy some energy range around the E_f_. Hence, no Dirac gap is opened. It is noted that Mn doped Bi_2_Se_3_ with in-plane tensile strain is metal transport, but Mn doped Bi_2_Se_3_ with out-of-plane tensile strain is semiconductor. This difference can well explain why the ferromagnetism in the case with in-plane strain is stronger than that with out-of-plane strain.

In summary, by using first-principles calculations, the magnetic properties and electronic structures of Mn doped Bi_2_Se_3_ with strain are explored. Our results firstly show that modest in-plane or out-of-plane tensile strains will make Mn doped Bi_2_Se_3_ become more stable. Then it is found that modest in-plane or out-of-plane tensile strains will make the ferromagnetism of Mn doped Bi_2_Se_3_ become stronger. Meanwhile, we find that out-of-plane tensile strain can modulates the Dirac gap and E_f_ of Mn doped Bi_2_Se_3_. This study provides a new route to high temperature QAHE.

## Methods

First-principles calculations were performed within the framework of DFT using the projector-augmented wave^37^ method as implemented in the Vienna ab-initio simulation package (VASP)^38^,^39^,^40^. Generalized gradient approximation (PBE-GGA) was adopted^41^ for treating the exchange correlation interactions. In our calculations we used a 2 × 2 × 1 slab supercell of Bi_2_Se_3_ with 6 quintuple layers (QLs) (Fig. 1a). The atomic structures were fully optimized until the Hellmann-Feynman forces on each ion were smaller than 0.02 eV/Å. A plane-wave energy cut-off of 400 eV, a *k*-point grid of 5 × 5 × 1, and the Gaussian smearing method with a smearing width of 0.1 eV were used integrations over the Brillouin zone. Unless mentioned other wise, spin-orbit coupling (SOC) and GGA+U^42^ calculations with U = 4.5 eV, J = 0.5 eV are taken into account in all calculations. The viability of OA and OB configurations is also estimated by a larger 3 × 3 × 1 slab supercell of 6QLs Bi_2_Se_3_(See Supplementary Fig. S2 and Table S2). Note that van der Waals (vdW) corrections^43^ is not involved in our calculations. But our test calculations indicate that vdW corrections do not change the conclusions of this study and only have some effects on exact value of our results (See Supplementary Table S3). To simulate out-of-plane (in-plane) strains, the out-of-plane (in-plane) lattice constants *c* (*a* and *b*) are elongated or compressed synchronously, then *a* and *b* (*c*) lattice parameters is adjusted to keep the cell volume constant, as treating in some studies^44^,^45^. In our simulations, percent strain means the change of the lattice. For each strain case, the atom positions are fully optimized with the same force criterion as that in the case without strain.

## Additional Information

**How to cite this article**: Qi, S. *et al*. Strain-modulated ferromagnetism and band gap of Mn doped Bi_2_Se_3_. *Sci. Rep.* **6**, 29161; doi: 10.1038/srep29161 (2016).

## Supplementary Material

Supplementary Information
