# Supplementary material for: Strain-modulated ferromagnetism and band gap of Mn doped Bi2Se3
Source: Sci Rep. 2016 Jul 4;6:29161. doi: 10.1038/srep29161 (PMC4931584; doi:10.1038/srep29161)
Supplement: Supplementary Information [file srep29161-s1.pdf]

Supplementary material for

Strain-modulated ferromagnetism and band gap of Mn doped Bi_2Se_3

Shifei Qi, Hualing Yang, Juan Chen, Xiaoyang Zhang, Yingping Yang & Xiaohong Xu

School of Chemistry and Materials Science, Shanxi Normal University, Linfen, Shanxi 041004, China

1. The estimation of T_c and magnetic anisotropy energy (MAE)

We have calculated the Curie temperature under different strains within the mean-field theory. On one hand, in the Heisenberg model within the mean-field approximation, the total energy difference ΔE between the ferromagnetic and antiferromagnetic states can be expressed as:

$$\Delta E = s^2 x^2 \sum_{i \neq 0} J_{0i} \ ,$$

where x is the concentration of the magnetic ions, s is the unit vector of magnetic moment, and i sums over all sites of the cation sublattices. Here, the energy difference ΔE can be directly obtained from the first-principles calculations. On the other hand, within the mean-field theory one can estimate the Curie temperature T_C by using the Brillouin function expression, known as the molecular field theory, leading to $k_B T_c = \frac{2}{3} s^2 x \sum_{i \neq 0} J_{0i}$, where k_B is the Boltzmann constant. By combing the above two equations, one can find that $k_B T_c = \frac{2}{3} \Delta E / x$, which allows for the evaluation of the Curie temperature T_C from the first-principles calculations.

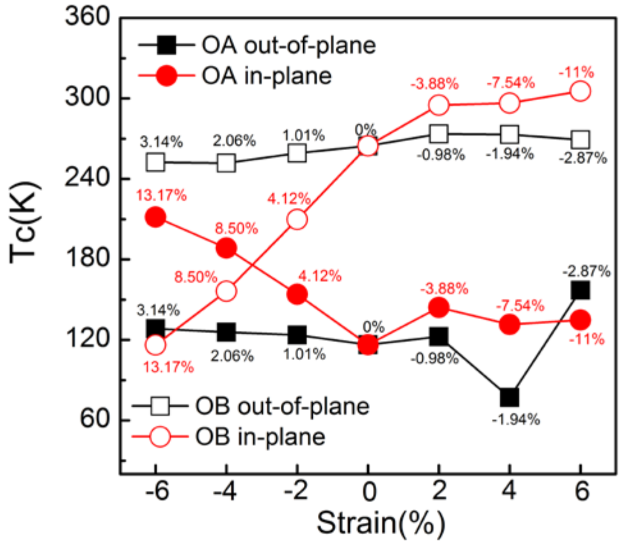

Figure S1. The Curie temperature and calculated values of out-of-plane or in-plane strains respectively in-plane and out-of-plane strains in OA and OB configurations.

Usually, the Tc from the mean-field theory is larger about 50% than that from Monte Carlo simulation [PRB, 79,235202,2009]. Hence the Tc of Mn doped Bi_2Se_3 with different strains should be in the range of 30-150 K at 4.16% Mn doping.

We have also calculated the magnetic anisotropy energy (MAE) of A and B configurations. As listed in the Table S1, the results show that out-of-plane ferromagnetism is more stable than in-plane one. This result agrees with experimental finding from Xu *et al.* [Nat. Phys. 8, 616, 2012].

Supplementary Table S1. The magnetic anisotropy energy (MAE) of the A and B configurations without strain.

configuration		In-plane	Out-of-plane
A	$\mu(\mu_B)$	2.01; 0; 0	0; 0; 2.01
	$MAE_{\parallel-\perp}$(meV)	0.549	
B	$\mu(\mu_B)$	1.88;0;0	0;0;1.88
	$MAE_{\parallel-\perp}$(meV)	5.668	

2. The estimation of viability for OA and OB configurations

In our calculations, we have to use a 2×2×1 supercell of 6QLs Bi_2Se_3 (including 120 atoms) considering of amount of computer time-consuming for lager supercell. A 2×2×1 supercell was also used in the similar theoretical calculations from the same reason [PRL, 109, 266405, 2013]. In fact, in our calculations, the estimated concentration of Mn doping is 4.16%, which is in the range of related experiment [Nat. Phys. 8, 616, 2012]. We also estimate the viability for OA and OB configurations using a larger 3×3×1 supercell of 6QLs Bi_2Se_3 (including 270 atoms) with the help

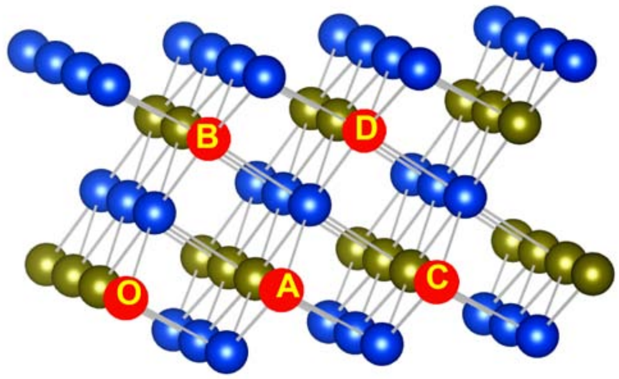

Supplementary Figure S2. 1 of 6QLs in a 3×3×1 Bi_2Se_3 supercell is used to clarity the doped configurations.

of 432 cores supercomputer. As shown in the Figure S2, we design four configurations: OA and OB (two doped Mn atoms are close), OC and OD (two doped Mn atoms are far). From the Table R2, we can find that the FM stability of OA and OB configurations are -32.96 and -74.95 meV from a 2×2×1 supercell (4.16% Mn doping). Employing of a larger 3×3×1 supercell (1.85% Mn doping), the FM stability of OA and OB configurations are -12.66 and -22.45 meV. The FM stability of OC and OD configurations are -15.53 and -4.32 meV. Hence, the FM state should be the most stable in different Mn doping concentrations, which is in agreement with related experiment [Nat. Phys. 8, 616, 2012]. From the Table S2, we can also find that the stability of four configurations is OD>OB>OC>OA. This indicates that doped Mn atoms are preferred to diluted distribution, which approves the estimation of this

reviewer. But, we also emphasis that the energy difference between OD and OA or OB configurations is only 20-75 meV. Thus, all doping configurations might appear in experiment. We think that the conclusions based OA and OB configurations should be true of OC and OD configurations. But we can not finish all calculations using a 3×3×1 supercell considering of quite amount of super computer time-consuming.

Supplementary Table S2. The relative stability of FM and AFM states for the OA and OB configurations without strain in different supercell.

**Conf.**	**2*2*1 (4.16%)**			**3*3*1 (1.85%)**		
	E_{FM}(eV)	E_{AFM}(eV)	$\Delta E_{FM-AFM}/2$(meV)	E_{FM} (eV)	E_{AFM}(eV)	ΔE_{FM-AFM} (meV)
OA	-248.98661	-248.92068	-32.96	-549.61100	-549.59834	-12.66
OB	-249.12333	-248.97344	-74.95	-549.65915	-549.63701	-22.45
OC	-	-	-	-549.61585	-549.60032	-15.53
OD	-	-	-	-549.68618	-549.68195	-4.32

3. The effect of vdW correction on our results

Supplementary Table S3. The influence of vdW correction on the relative stability of FM and AFM states for the OA and OB configurations without strains.

**Conf.**	**No vdW**			**With vdW**		
	E_{FM}(eV)	E_{AFM}(eV)	$\Delta E_{FM-AFM}/2$(meV)	E_{FM} (eV)	E_{AFM}(eV)	$\Delta E_{FM-AFM}/2$(meV)
OA	-248.98661	-248.92068	-32.96	-247.81920	-247.75584	-31.68
OB	-249.12333	-248.97344	-74.95	-247.90654	-247.85705	-24.74

4. Estimation of stability for the Mn doped Bi_2Se_3 under 6% out-of-plane tensile strain。

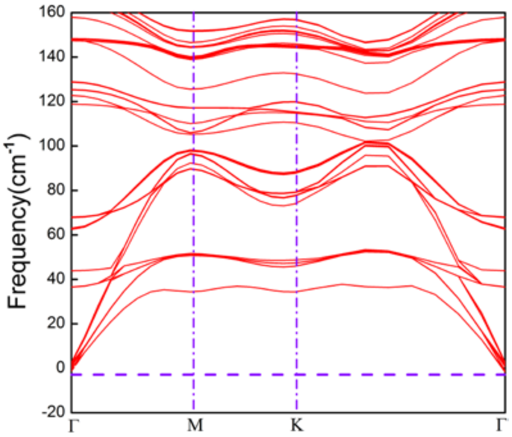

Supplementary Figure S3. Phonon dispersion curves of Mn doped Bi_2Se_3 under 6% out-of-plane tensile strain calculated from a 6QLs 1×1 supercell.
